# Wildfire risk to United States cultural resources

**DOI:** 10.1038/s41598-026-52407-9

**Published:** 2026-05-12

**Authors:** Mona M. Farnisa, Benjamin S. Halpern, Caitlin R. Fong

**Affiliations:** 1 National Center for Ecological Analysis and Synthesis, University of California Santa Barbara, Santa Barbara, CA 93106 USA; 2 Bren School of Environmental Science, University of California Santa Barbara, Santa Barbara, CA 93106 USA

**Keywords:** Cultural resources, Heritage resources, Structure loss, Wildfire, National register of historic places, Hazard, Environmental social sciences, Environmental studies, Geography, Geography, Natural hazards

## Abstract

Wildfire research and management typically emphasize risks to ecosystems and infrastructure, especially homes. Yet, communities and countries designate certain places and structures as culturally important for all; because they are often immovable and irreplaceable, they are uniquely at risk from wildfire. We present the first national-scale assessment of wildfire risk to cultural heritage assets in the U.S. Here, wildfire risk is defined as the spatial coincidence of cultural heritage resources and modeled wildfire hazard. Our analysis integrates high-resolution burn probability models from the Fire Simulation project with spatial data for 56,103 National Register of Historic Places (NRHP). Places were further assessed by type, reflecting potential cultural loss if destroyed, including buildings, districts, structures, objects, and sites. Places were also categorized by cultural significance (local, state, national) to evaluate how risk varies across levels of importance. Risk was concentrated in the western United States, with hotspots in the Southeast. Buildings and districts comprise 90% of listed resources and may be more vulnerable because their value depends on physical form; other resource types may be less vulnerable. Only 36% of exposed places are nationally significant; most are state or locally significant. By identifying where and what is most at risk, this study provides a foundation for proactive planning to safeguard the places that anchor community identity, collective memory, and national heritage before they are permanently lost.

## Introduction

Wildfire frequency, intensity, and extent have increased substantially in the United States (U.S.) over the past four decades, driven by climate change, land-use shifts, and expansion of the wildland–urban interface^1–3^. These changes have resulted in rising structural losses nationwide, with tens of thousands of buildings destroyed annually and increasing areas burned across diverse ecosystems, from the western U.S. to the Great Plains and eastern temperate forests^4–8^. Although the loss of any resource can be tragic, collectively as a community and society we have designated some places and structures as culturally significant, whose loss could be irreplaceable. The shift in fire regime underscores the urgent need to assess exposure and vulnerability of these cultural heritage resources to wildfire hazard.

Locations, structures, and objects that embody cultural, historical, or social value and are passed down through generations are broadly referred to as cultural heritage resources^9,10^. Preserving cultural, historical, and archaeological resources safeguards a nation’s shared history and strengthens a sense of identity and connection across generations. These resources contribute to our sense of place and identity, and are widely viewed as irreplaceable and worthy of preservation^11^. The global importance of cultural heritage and historical preservation was formally recognized in 1972, when UNESCO held the World Heritage Convention to protect and secure important cultural heritage resources and natural places^12^.

Cultural heritage resources can be highly vulnerable to wildfire due to their immovability and the use of traditional building materials, such as timber, with limited fire resistance in their original designs^13–15^. Most structures were not built to withstand fire and typically do not meet modern fire codes and standards^16^. Even stone buildings can suffer structural damage under extreme heat, including breakage, microfracturing, and weakened integrity^14,17,18^. Smaller objects, by contrast (e.g., paintings, sculptures, manuscripts, coins, etc.), can often be relocated either temporarily or permanently, and are thus more easily protected. Beyond their physical loss, the destruction of historic and cultural heritage resources carries a social cost, erasing evidence of a community’s shared history and cultural identity (Jones and Leech, 2015; Wells, 2010). If these resources are lost to wildfire, it is unclear whether they can ever be replaced, highlighting the importance of identifying vulnerable sites and prioritizing investments for their protection.

Despite widespread recognition of the importance of protecting cultural heritage resources, national assessments of wildfire risk to these resources are rare. Indeed, wildfire’s effect on cultural heritage resources remains underexamined globally^10^. In the U.S, existing studies are limited to regional cases, such as hazard assessments in Idaho, Montana, and Louisiana^21,22^. This limited scope of work is paralleled in other countries, such as Spain, Greece, Portugal, and China, where there are sub-national assessments of wildfire threats to cultural heritage resources, but no consistent national assessment^23–27^. Indeed, only Italy has a national-level assessment of wildfire risk to cultural heritage^28^. Collectively, these patterns reveal a critical gap: while the threat is clear, the U.S. lacks a systematic assessment to identify at-risk resources to guide prioritization.

Here, we present the first national-scale assessment of wildfire risk to cultural heritage resources in the U.S. We operationalize risk as the spatial intersection of wildfire hazard—represented by burn probability—and the distribution of cultural resources. In this framework, exposure is treated as a binary filter (i.e., whether a resource is located within the modeled domain). A comprehensive, national assessment is essential to inform targeted preservation efforts, guide allocation of mitigation resources, and support strategic planning for protecting cultural heritage against future wildfires.

## Results

**Fig. 1:**
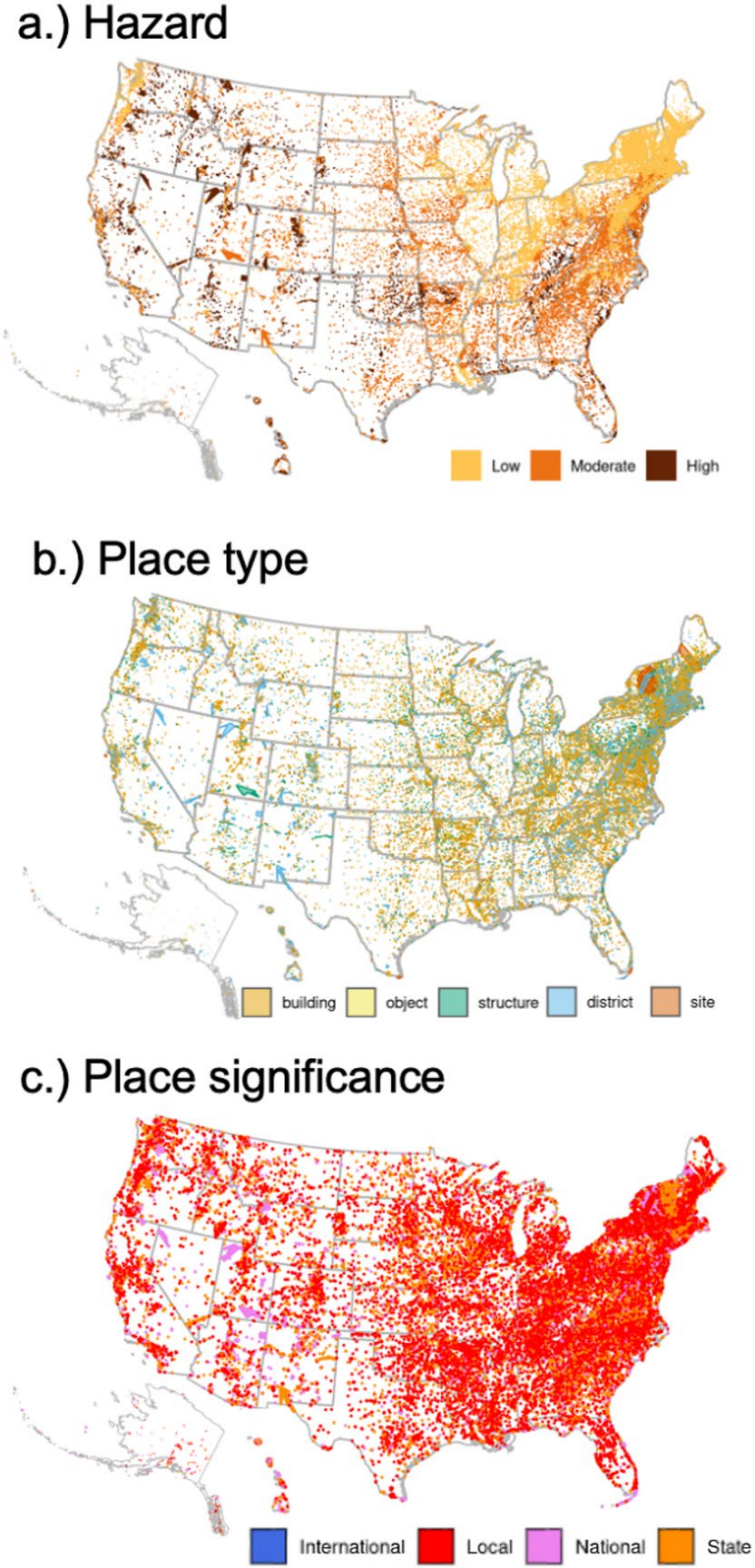
Spatial distribution of wildfire hazard and place-type classification for places in the U.S. National Register of Historic Places (NRHP). (**a**) Wildfire hazard categorized into burn probability bins. (**b**) Place-type classification represented by place category (building, district, structure, object, or site) as well as level of significance (local, state, national, international). Alaska is shown at 50% scale.

National Resource Heritage Places (NRHP) (hereafter called places) are unevenly distributed across the U.S. Although the eastern U.S. contains more NRHP places overall, these areas generally coincide with lower wildfire hazard (Fig. 1a). High hazard concentrations begin in the Rocky Mountains, with additional hotspots in Oklahoma, the Appalachians, and along the southeastern coast (Fig. 1a). The types of historic resources (buildings, districts, objects, sites, or structures) as well as level of significance (local, state, national) are relatively consistent across regions (Fig. 1b,c), though notably more concentrated in the east.

Across 56,103 NRHP records, 27 were not classified into the place type categories and were excluded from subsequent analysis. Among the remaining 56,076 classified places, buildings are by far the most common type (68%), followed by districts (21%), structures (6%), sites (3.6%), and objects (0.4%) (Table 1). The majority of places fall in the low- and moderate-hazard classes, consistent with the percentile thresholds used to define burn probability bins (0–50th, 50–90th, and 90–100th percentiles). However, hazard class varied significantly by place type (χ^2^ = 460, *p* < 0.0001), where sites and, to a lesser extent, structures are disproportionately represented in the high-hazard category, resulting in increased risk to these place types.

**Table 1:** Summary of wildfire hazard classification by place type. Each cell shows observed counts, with expected counts under independence shown in parentheses (rounded to the nearest whole number). Expected counts were calculated from marginal totals assuming independence between hazard class and place type. Hazard class varied significantly by place type (χ^2^ = 460, *p* < 0.0001). Note that 27 NRHP places were not classified into the five place type categories shown and are excluded from this analysis.

		Hazard bin		
		Low	Moderate	High
Place type	Building	18,961 (19,098)	15,651 (15,280)	3587 (3821)
	District	6631 (6074)	4438 (4860)	1080 (1215)
	Structure	1526 (1723)	1394 (1378)	526 (345)
	Object	119 (122)	96 (98)	29 (24)
	Site	800 (1,020)	853 (816)	388 (204)

**Fig. 2:**
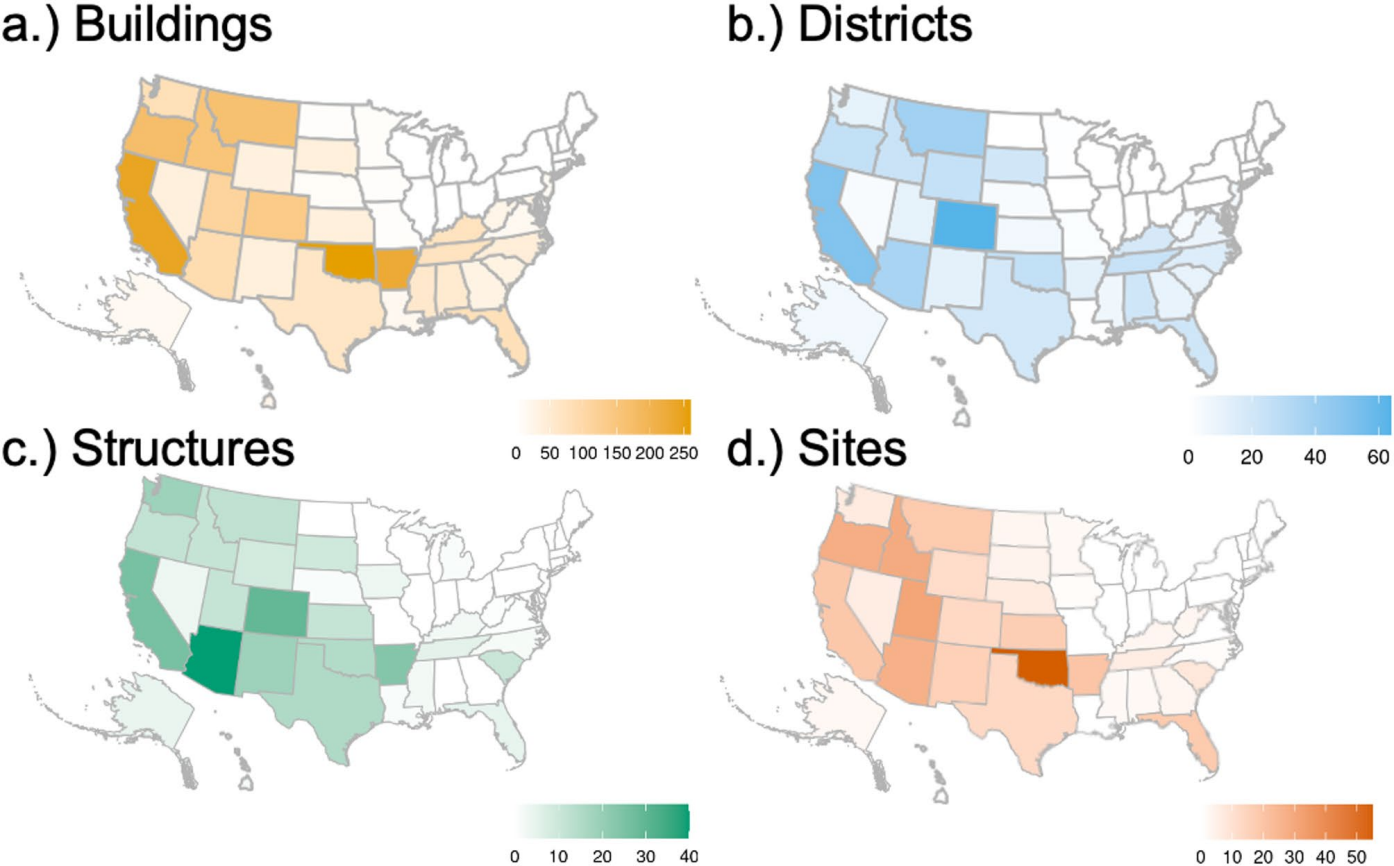
State-level maps of high-hazard NRHP places by place category. (**a**) Buildings. (**b**) Districts. (**c**) Structures. (**d**) Sites. Each panel uses an independent scale for high-hazard counts to reflect differences in place distribution across types. Because objects are rare (*n* = 29), they are not mapped. Alaska is shown at 50% scale.

High-hazard NRHP places (top 10% of burn probability) are distributed across nearly all states, but their concentrations vary by place type (Fig. 2). Among buildings, the largest counts occur in California, Oklahoma, and Arkansas (Fig. 2a). Secondary clusters appear across the Intermountain West, including Oregon, Idaho, and Montana. Districts show their strongest concentrations in Colorado, followed by California and Montana (Fig. 2b). Structures are likewise most common in Colorado and Arizona, with smaller clusters in California and Arkansas (Fig. 2c). Sites exhibit a distinct spatial pattern, with a strong concentration in Oklahoma (Fig. 2d). Objects are rare (*n* = 29) and not mapped, with the number per state ranging from 0 to 6. These contrasts highlight that even within the limited set of high-hazard places, place-types vary geographically to shape wildfire risk to cultural resources.

When disaggregated by significance, NRHP places are dominated by local designations (68%), followed by state (24%) and national (8%) significance (Table 2). High-hazard places are slightly more concentrated among nationally significant sites (15%) than state- (11%) or locally significant (9%) sites, highlighting that the most important cultural resources are disproportionately exposed to wildfire (χ^2^ = 226, *p* < 0.0001).

**Table 2:** Summary of wildfire hazard by level of significance. Observed counts are shown with expected counts under independence in parentheses (rounded to the nearest whole number). One international site is excluded. Hazard class varies significantly by significance level (χ^2^ = 226, *p* < 0.0001). A total of 111 NRHP places were not assigned a significance category and are excluded from analysis.

		Hazard bin		
		Low	Moderate	High
Level of significance	National	2233 (2213)	1532 (1771)	661 (443)
	State	6355 (6717)	5586 (5375)	1495 (1345)
	Local	19,402 (19,061)	15,281 (15,253)	3447 (3816)

Within the high-hazard class, NRHP place types are unevenly distributed across degrees of significance (χ^2^ = 539, *p* < 0.0001). High hazard buildings are disproportionately of local significance (70%, Table 3). Districts and structures show the same general pattern but at lower percentages. Objects are extremely rare among high-hazard places, and most are of state-level significance. A notable exception to this trend is sites, which show a relatively greater share of national significance (37%) compared to other place types, which skew local. This suggests that immovable cultural heritage landscapes such as battlefields or archaeological sites are more often nationally designated.

**Table 3:** Summary of high-hazard places by type. Each cell shows observed counts, with expected counts under independence shown in parentheses (rounded to the nearest whole number). High-hazard places are shown by level of significance (National, State, Local) and place category (building, district, structure, object, site). Hazard class is significantly associated with both place type and significance level (χ^2^ = 539, *p* < 0.0001).

		High hazard bin		
		National	State	Local
Level of significance	Building	233 (422)	825 (955)	2522 (2202)
	District	193 (127)	341 (288)	546 (664)
	Structure	87 (62)	203 (140)	236 (324)
	Object	6 (3)	15 (8)	8 (18)
	Site	142 (46)	111 (103)	134 (238)

**Fig. 3:**
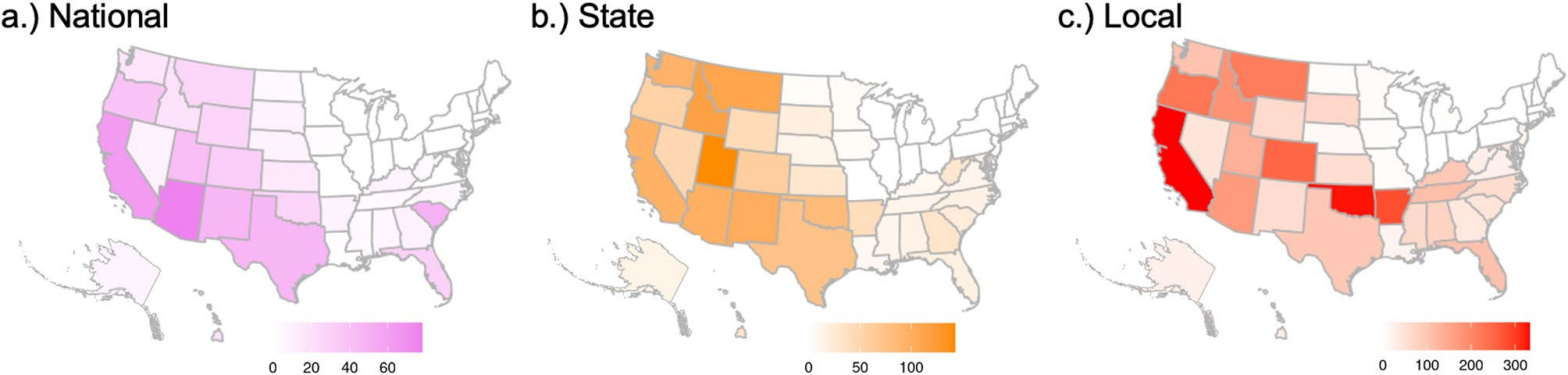
State-level maps of high-hazard NRHP places by level of significance. (**a**) National significance. (**b**) State significance. (**c**) Local significance. Each panel uses an independent scale for high-hazard counts to reflect differences in place distribution across significance levels. Alaska is shown at 50% scale for visibility.

The distribution of high-hazard places varies across states and by significance, indicating that prioritization strategies may need to differ depending on whether federal, state, or local agencies are responsible for their protection (Fig. 3). Nationally significant high-hazard places are spread across many western states but are disproportionately concentrated in Arizona (*N* = 78). By contrast, Utah contains the most state-significant places at high hazard (*N* = 143), while California and Oklahoma have the largest numbers of locally significant places at high hazard (*N* = 333 and 328, respectively). Arkansas is also unexpectedly high, with a high count of local-level high-hazard places, despite generally lower wildfire profiles (*N* = 281).

## Discussion

Our analysis provides a national assessment of wildfire risk to U.S. cultural heritage resources, identifying priorities for preservation, mitigation, and adaptation across federal, state, and local jurisdictions. High-risk cultural heritage resources are concentrated in the West, with additional emerging hotspots in the southeastern and south-central states, highlighting regions where urgent action is needed. This spatial distribution mirrors historical patterns of structural loss, with most destruction occurring in western states, particularly California, Oregon, and Colorado^8^ The southeastern and south-central hotspots suggest new areas of concern^6^, warranting greater attention from state and regional preservation programs. Climate change, with its associated increases in temperature, drought frequency, and extreme fire weather, is likely to intensify these trends, expanding hazard zones and elevating the exposure of cultural heritage sites in both traditionally high-risk and previously lower-risk regions^29–33^. Thus, while our analysis provides a snapshot of current patterns, shifting wildfire dynamics mean that new areas may increasingly require mitigation and preservation attention in the future.

Historic buildings and districts dominate cultural heritage resources in the U.S., and these types of places may face uniquely high wildfire risk because they are immovable and their cultural heritage value is tied to the physical structure, such that their loss represents permanent cultural heritage loss^18^. Mitigation must therefore occur on-site, with strategies spanning vegetation and fuel management, structural hardening, and suppression capacity. Defensible space and vegetation management are often key, though challenges arise when the surrounding landscape itself holds cultural value. For example, at The Ahwahnee Lodge in Yosemite National Park, the historic character of the landscape depends on specific vegetation features, including wooded evergreen entrance corridors, screening trees around parking areas and tennis courts, landscaped shrubs, and dense tree screening along the site’s perimeter^34^. Altering or removing these elements for wildfire mitigation could compromise the historic integrity of the site, illustrating the need for carefully balanced strategies.

Many traditional approaches to creating fire-safe structures and buildings that rely on modern materials and building codes are difficult to implement for most cultural places. The Secretary of the Interior’s Standards for the Treatment of Historic Properties (36 CFR Part 68) provide guidance on preserving, rehabilitating, restoring, and reconstructing historic properties, offering guardrails for interventions that maintain a building’s historic character while allowing for context-sensitive adaptations. These Standards emphasize evaluating potential hazards, maintaining structures in good condition, and documenting historic features, which creates a framework for integrating wildfire resilience into historic treatments. Performance-based design offers a flexible framework for structural hardening, focused on the goal and outcome of fire safety rather than prescriptions from the latest building codes (e.g.,^35,36^. For example, when undertaking rehabilitation, extensively deteriorated or damaged features can be replaced with fire-resistant materials or compatible substitutes. During preservation and restoration, existing coatings, barriers, or protective layers can be maintained or upgraded to resist ignition. When performing reconstruction, modern hardening strategies such as ember-resistant roofing or fire-rated assemblies can be incorporated in ways that visually align with the historic design. Additionally, less visible interventions can enhance safety without compromising historic integrity. For example, concealed sprinkler systems, hidden piping, and wireless fire detection systems can be installed to protect structures from wildfire while remaining unobtrusive^37,38^. By integrating wildfire considerations into treatments, owners and preservationists can reduce the risk of catastrophic loss while maintaining the historic and cultural significance of the property. Thus, effective on-site mitigation requires collaboration between fire engineers, preservationists, and local authorities to balance risk reduction with conservation goals^18^. These approaches highlight that preventing the loss of immovable cultural heritage requires active, context-specific interventions, rather than reliance on relocation or compliance with building codes.

The loss of historic properties to wildfire is a growing concern, but relatively few well-documented cases exist, limiting our understanding of potential consequences for cultural heritage. Nearly 2500 properties have been delisted from the National Register of Historic Places, but the reasons for removal are often not specified in the database, making it difficult to quantify losses specifically due to wildfire. What is clear is that wildfire can completely destroy the physical structure of a historic property and erase the features that give it cultural and historical significance. Recent events in 2025 illustrate these risks and their emerging consequences. In January of 2025, the Will Rogers Ranch House and the Andrew McNally House in Los Angeles were lost to the Palisades and Eaton fires, respectively. There are plans underway to rebuild portions of the Will Rogers property, including the park visitors center, at a projected cost of $3.6 million from the state and an additional $1.5 million from conservation agencies^39^, with key historic elements that were evacuated now guiding reconstruction. However, the fate of the McNally House remains unclear. The Grand Canyon Lodge originally built in 1927, was destroyed in the Dragon Bravo wildfire that burned in Grand Canyon National Park in July 2025. The lodge had burned before in 1932 and was rebuilt; however, at this moment it is unclear if there is appetite for another rebuild^40^. In each of these examples, the buildings were completely lost, and it is uncertain whether any reconstruction efforts will meaningfully restore the cultural and historic value they represented.

While federally significant places may dominate national preservation efforts, the majority of NRHP places are state- and locally- significant. Thus, our assessment provides a valuable resource for regional authorities to prioritize mitigation and protection strategies. Only a handful of states, such as California through CAL FIRE’s Cultural Resources Management Program, have formal initiatives to address wildfire threats to cultural heritage resources, and most states lack dedicated risk assessments. Thus, in most states, cultural heritage considerations are not systematically incorporated into dedicated wildfire planning frameworks and instead must be integrated through broader all-hazards planning processes. Our results provide a standardized, nationally consistent dataset that can support state and regional agencies in pre-incident planning and mitigation prioritization. Within this planning context, cultural heritage information functions as one component of multi-objective fire planning frameworks, alongside other values at risk such as infrastructure, ecological assets, and community protection priorities. While life safety and critical infrastructure remain the primary determinants of operational and tactical decision-making, cultural heritage resources may be considered within pre-incident planning and prioritization frameworks when evaluating multiple competing values at risk. For example, within a given state, decision-makers could rank properties by burn probability, vulnerability, or significance, creating tailored priority lists that reflect both hazard and cultural heritage value. These rankings could be further refined through participatory approaches, where local communities identify places of cultural or historical importance that may not be fully captured by federal designations (e.g.,^41–44^, demonstrating how combining technical risk assessments with community knowledge can yield more inclusive and context-specific prioritization. Applying such methods in the U.S. would ensure that preservation strategies align not only with hazard exposure but also with local values, thereby enhancing their legitimacy and effectiveness.

Although NRHP dataset provides a valuable foundation, it lacks detailed site-specific contextual data, highlighting the need for further research to refine wildfire risk assessments for cultural heritage resources. Our national-to-regional risk assessment offers a broad overview, but cannot account for localized mitigation measures. For instance, the Historic Annapolis Yacht Club in Maryland and Christ Church in Philadelphia have undergone fire sprinkler retrofits to enhance safety while preserving historic integrity^45^. Similarly, the Getty Center in Los Angeles employs fire-resistant materials and landscaping to protect its collections during wildfires^46^. However, such measures are likely less common in less nationally significant properties, though comprehensive data on their prevalence and effectiveness is scarce. Therefore, while our assessment provides valuable information, more site-specific evaluations are essential to develop targeted and effective wildfire mitigation strategies for cultural heritage sites.

While this study provides a spatially consistent assessment of wildfire exposure for formally designated cultural heritage resources in the United States, it is important to recognize three key limitations that affect interpretation. First, the analysis is limited to physically mappable and formally documented resources in the NRHP. As a result, it does not capture the full breadth of cultural heritage, including undocumented sites, iconic species, and access to cultural practices^47–50^. This introduces a systematic bias toward formally recognized, physically bounded heritage, which may underrepresent culturally significant places associated with communities whose heritage is less consistently documented or formally designated. Second, our analysis is constrained to places formally registered with the U.S. federal government, which may result in underrepresentation of different types of heritages. This is consistent with broader critiques suggesting that formal heritage registration systems tend to reflect the values of dominant groups, systematically marginalizing the heritage of non-dominant communities^51^. Finally, while the model quantifies exposure to wildfire hazard, it does not and cannot translate that exposure into the magnitude or nature of cultural loss. Consequently, the results should not be interpreted as measuring the “value” or “impact” of potential loss, but rather as identifying where formally recognized heritage assets intersect with modeled wildfire hazard. Understanding the cultural consequences of such losses requires qualitative, historical, and community-based perspectives that extend beyond the scope of spatial hazard modeling^52^.

This study provides a nationwide assessment of wildfire risk to U.S. cultural heritage resources, revealing high-risk regions and highlighting the potential vulnerability of historic buildings and structures, which are immovable and can be permanently lost. Moreover, the open-source workflow developed here can be applied to other national hazard assessments or adapted for finer-scale regional analyses, providing a consistent methodology for prioritizing and comparing risk across cultural heritage places. Our work complements existing efforts, such as National Park Service funded projects to evaluate wildfire threats for select historic structures within individual parks in the west (National Park Service Project: Develop Treatments for Cultural Resources Threatened by Wildland Fire at Pacific West Parks) and California’s state-level programs for protecting cultural heritage resources (CAL FIRE: Cultural Resources Management Program). By creating a standardized, national-scale assessment, this study provides a resource that enables comparisons across sites, informs targeted preservation strategies, and helps decision-makers at federal, state, and local levels prioritize mitigation efforts to safeguard cultural heritage from wildfire threats.

## Methods

In the U.S., the National Register of Historic Places (NRHP) is the official federal list of cultural heritage resources (hereafter referred to as places) recognized for their cultural, historical, or archaeological importance. Cultural heritage resources include buildings, structures, districts, sites, and objects. Buildings are constructed primarily to house or shelter human activity, such as residences. Structures are functional constructions intended for purposes other than human shelter, such as bridges or dams. Districts encompass areas containing a significant concentration of historically or aesthetically connected buildings, structures, sites, or objects. Objects are typically artistic or commemorative, often movable, and associated with a particular setting, such as monuments or sculptures. Sites represent locations whose significance does not depend on a standing building or structure, such as battlefields. Places are eligible to be listed if they meet at least one of four criteria: association with significant historical events, association with significant historical persons, architectural or artistic distinction, or potential to yield important archaeological information^53^. The NRHP lists a total of 100,117 places across the 50 states and territories.

We assess wildfire risk to cultural resources as the spatial co-location of National Register of Historic Places (NRHP) resources with modeled wildfire hazard derived from FSim burn probability surfaces. In this framework, risk is operationalized as hazard exposure rescaled between 0 and 1 rather than a composite function of independent components. The NRHP also categorizes places by type—buildings, districts, structures, objects, and sites—as well as their level of significance—local, state, national, or international. While these categories were not incorporated into a composite risk calculation, we use it as an informative way to filter, aggregate, and visualize the data to aid interpretation.

### Exposure

Exposure refers to the spatial location of cultural heritage resources; if we could not identify the location of the place, we could not map its risk to wildfire. We mapped locations by joining the *2025 NRHP Listed Properties dataset* (*N* = 100,117) with the accompanying *2024 spatial dataset* of place locations (available as downloads through nps.gov). Records lacking spatial geometry, for example sensitive archaeological sites, were excluded (*N* = 9096). Duplicate entries (*N* = 4259) resulted from multiple updates, edits, or geometries associated with the same property ID; for each duplicate, we retained the most recently modified record based on creation, edit, and listing dates. An additional 39 locations fell outside the U.S. land boundary (e.g., offshore lighthouses, shipwrecks, or sites in territories without wildfire hazard data) and were removed. The resulting dataset consisted of 71,630 points and 19,354 polygons. To ensure a spatial footprint for hazard calculations, points were buffered by 30 m to match our hazard model raster (see Hazard below), while polygons were retained as-is. The buffered points and polygons were then combined into a single spatial layer. Sites with zero wildfire hazard (*N* = 31,939) were subsequently removed, leaving 56,103 places for assessment. All remaining places were assigned an exposure value of one, indicating they would be exposed if a wildfire occurred at that location. As such, exposure is treated as binary and uniform across all retained locations, so it does not differentiate risk outcomes. Notably, there is only one international site, which we drop from subsequent analysis and mapping.

### Hazard

We quantified wildfire hazard using the U.S. Forest Service’s Fire Simulation model (FSim), a high-resolution fire simulator that produces annualized burn probabilities (BP) at 30 m resolution, allowing fine-scale hazard estimation for individual places. FSim incorporates historical fire occurrence, weather, terrain, and fuel conditions, generating probabilistic fire outcomes across the landscape. Notably, FSim burn probabilities are calibrated to historical fire weather conditions and do not capture projected changes in fire climate. Importantly, FSim provides a nationally consistent assessment, enabling comparisons across the entire U.S. Data layers are publicly available from the Wildfire Risk to Communities v2 (2024) dataset (Scott et al. 2024) (https://www.wildfirerisk.org). BP values across the entire U.S., including regions without any cultural heritage places, ranged from 0 to 0.14, indicating, in the most extreme locations, a 14% annual burn chance within that pixel, equivalent to roughly one fire every seven years. The BP raster was produced using vegetation and wildland fuel data from LANDFIRE 2020 (v2.2.0) and land cover and disturbance conditions up to the end of 2020.

Because wildfire hazard can extend beyond a place’s footprint, each place was buffered by 500 m and 2000 m to account for short- and medium-range ember transport^55–59^. Burn probabilities (BP) from the FSim raster were extracted within these buffers to quantify hazard for each place. We extracted the mean and maximum BP to capture both average condition and the most extreme scenario. Pairwise t-tests comparing values for the two buffer distances for either mean and maximum BP indicated statistically significant but marginal differences (effect sizes = − 0.0005 and − 0.0009), indicating either buffer range would produce the same results. We used the 500 m buffer because while long-range spotting is possible, the probability of ignition drops sharply with distance, so the 500 m buffer should capture the bulk of realistic hazard^60–62^.

After calculating the composite BP for the buffer around each place, we rescaled values to a 0–1 range using the 99th percentile as the upper limit. This rescaling was done to reduce the influence of extreme outliers and facilitate comparison across places, ensuring that very high BP values do not disproportionately dominate the hazard metric. Both the mean and maximum BP within the 500 m buffer around each place were considered, and hazard was calculated as the average of the rescaled mean and maximum. Averaging these two measures captures both the typical wildfire hazard across the surrounding area (mean) and the peak hazard at any point within the buffer (maximum), providing a balanced representation of potential hazard.

We categorized hazard into three levels—High, Moderate, and Low—based on the 90th and 50th percentiles. BPs were highly left-skewed, reflecting that most places are located in areas with low wildfire hazard. To quantify how place type and level of significance varied by hazard bin, we performed a series of chi-square tests.

To evaluate the robustness of our hazard classification, we tested alternative measures of burn probability. We compared classifications derived separately from normalized mean or maximum burn probability to the composite bins using cross-tabulations. Both approaches showed high agreement with the composite classification (mean-based = 0.88; max-based = 0.94), with the maximum showing the strongest concordance, consistent with the notion that wildfire hazard is dominated by extreme exposure. Additionally, mean and maximum burn probabilities are highly collinear (linear regression: R^2^ = 0.934, *p* < 0.0001), justifying the use of the combined metric in subsequent analyses.

### Software

We used R (RStudio) R version 4.4.0 and Python (VS Code) version 3.10.12. R analyses relied on the following packages: tidyverse, sf, rnaturalearth, rnaturalearthdata, RColorBrewer, scales, tidycensus, ggpubr, biscale, dplyr, ggplot2, and *minpack.lm*. Python analyses relied on the following packages: geopandas, pandas, osmnx, networkx, numpy, matplotlib, rasterio, rasterstats, shapely, tqdm, tenacity, requests, concurrent.futures, multiprocessing, zipfile, io, logging, glob, json, csv, ast, signal, functools, and mpl_toolkits. For full documentation, including complete code required to reproduce analyses and figures, see https://github.com/WRI-Science/wildfire-risk-to-iconic-places-sci-rep

## Data Availability

All data are publicly available. NRHP can be found at https://www.nps.gov/subjects/nationalregister/data-downloads.htm while FSim data can be found at https://www.wildfirerisk.org. Note, these are living datasets, and the analyses will change as data are updated. In our GitHub release are the data associated directly with this manuscript https://github.com/WRI-Science/wildfire-risk-to-iconic-places-sci-rep.
